# Quality of life associated with treatment adherence in patients with type 2 diabetes: a cross-sectional study

**DOI:** 10.1186/1472-6963-8-164

**Published:** 2008-07-30

**Authors:** Yolanda V Martínez, Carlos A Prado-Aguilar, Ramón A Rascón-Pacheco, José J Valdivia-Martínez

**Affiliations:** 1 Unidad de Investigación Epidemiológica y en Servicios de Salud, Hospital General de Zona N° 1, Instituto Mexicano del Seguro Social, Aguascalientes, Aguascalientes, México; 2 Unidad de Investigación Epidemiológica y en Servicios de Salud, Instituto Mexicano del Seguro Social, Hermosillo, Sonora, México

## Abstract

**Background:**

Despite certain contradictions, an association has been identified between adherence to drug treatment and the quality of life in patients with type 2 diabetes. The contradictions observed emphasize the importance of using different methods to measure treatment adherence, or the association of psychological precursors of adherence with quality of life. For this reason, we have used an indirect method to measure adherence (pill count), as well as two adherence behaviour precursors (attitude and knowledge), to assess the association between adherence and the quality of life in type 2 diabetes patients.

**Methods:**

A cross-sectional comparative study on a random sample of 238 type 2 diabetic patients was carried out over one year in four family medicine units of the Mexican Institute of Social Security (IMSS) in Aguascalientes, Mexico. Treatment adherence was measured using the indirect method of pill count to assess adherence behaviour, obtaining information at two home visits. In the first we recorded the medicine prescribed and in the second, we counted the medicine remaining to determine the proportion of the medicine taken. We also assessed two adherence behaviour precursors: the patients' knowledge regarding their medical prescription measured through a structured questionnaire; and attitudes to treatment adherence using a Likert scale. Quality of life was measured through the WHOQOL-100 (the WHO Quality of Life questionnaire). Information concerning both knowledge and attitude was obtained through interviews with the patients. A multiple linear regression model was constructed to establish the relationship between each quality of life domain and the variables related to adherence, controlling for covariates.

**Results:**

There was no association between quality of life and treatment adherence behaviour. However, the combination of strong knowledge and a positive attitude was associated with five of the six quality of life domains.

**Conclusion:**

The results suggest that it is important to explore psychological precursors of treatment adherence behaviour in type 2 diabetic patients. Indeed, we consider that it will be useful to carry out interventions that change negative attitudes towards treatment adherence and that promote medical prescription knowledge, which may help to improve the quality of life of such patients.

## Background

When patients with diabetes do not adhere to their drug prescriptions the efficacy of the medication declines [1], as does their control of glycaemia. Accordingly, there is a higher risk of acute and chronic complications [2,3], leading to unnecessary hospital admissions [4]. A systematic review of adherence to diabetes medication reported frequencies between 36% to 93% [5]. Such variation was in part associated with the method used to determine adherence and indeed, while the adherence rate for patients taking sulfonylurea was 74.5% using electronic monitoring, self-reported adherence was 92.4% [6].

It is clear that in terms of quality of life, chronically ill individuals had lower mean quality of life domain scores when compared to healthy adults or childbearing women [7]. There are different results regarding the association between quality of life and glycemic control, and while some studies have clearly shown such an association [8-10], this is not evident in other studies [11-14]. It has been suggested that adherence to drug treatment and quality of life are linked, although some contradictory results have again appeared in different studies [15-19]. Some of these discrepancies may reflect the limitations of the different methods or instruments used to measure adherence. Therefore, it seems to be important to measure treatment adherence behaviour as well as its psychological precursors.

The objective of this study was to assess the association between quality of life and treatment adherence behaviour, medical prescription knowledge, attitude toward treatment adherence, and a combination of knowledge and attitude in type 2 diabetic patients.

## Methods

### Study design

A cross-sectional comparative study was carried out between March 2003 and March 2004 in four Family Medicine Units (FMU) of the Mexican Institute of Social Security (IMSS) in Aguascalientes, Mexico.

### Setting

In Mexico, the social security (IMSS) is the principal health service provider and offering medical services to more than 50% of the Mexican population. The IMSS also maintains a Register of Chronic Degenerative Patients (RPCD), a database that in 2001 had approximately 27,850 patients with diabetes in Aguascalientes. In this city, the IMSS has six FMU, with a total of 86 medical offices. Our study was carried out in 82.5% of the medical offices.

### Study population

Individuals that were included in this study were diagnosed with type 2 diabetes at least one year previously, they were over 40 years of age and underwent treatment with oral hypoglycemic agents. Patients with chronic complications or disabling diseases, and patients on insulin treatment were not included.

The sample size was calculated in order to identify a mean difference in the quality of life of 10 points (on a scale of 0 to 100), with a mean of 70 in patients that adhered to diabetes medication and a mean of 60 in those who did not, as well as a standard deviation of ± 23, an alpha coefficient of 0.05 and a power of 0.80. Accordingly, the sample size required was calculated to be 84 patients per group. This study was approved by the scientific and ethical review Committee N° 101 of the IMSS with reference number 2002/01/02/04 and written informed consent was obtained from all patients as they were recruited at the health care units. Subjects were randomly selected from the RPCD.

### Study variables

The dependent variable was the quality of life score, the independent variables were the Treatment Adherence Behaviour, Medical Prescription Knowledge, Attitude toward Treatment Adherence, and a combination of Knowledge and Attitude. Age, gender, educational level, marital status, hypertension, hypoglycaemic prescription, diabetes duration, fasting glucose, and HbA1c were used as control variables.

The Health Related Quality of Life (HRQOL) scale was defined as the individuals' perception of their situation in the context of the culture and value systems in which they live, and in relation to their goals, expectations, standards and concerns. This is a broad concept affected in a complex way by the individuals physical health, psychological state, level of independence, social relationships, personal beliefs and their relationship to salient features of their environment [20].

In 2001, the WHO organized a meeting to deal with the issue of "adherence". From the definitions of Haynes [21] and Rand [22], they adopted the definition of "adherence to long-term therapy" as: the extent to which a person's behaviour in taking medication, following a diet and/or executing lifestyle changes, corresponds with the agreed recommendations of a health care provider [23]. It is known that in order to achieve satisfactory adherence patients must possess adequate knowledge about self-care behaviour [24], and that attitudes play an important role in changing adherence behaviour, since they predict and explain human behaviour [25]. Therefore, we decided to measure Medical Prescription Knowledge and Attitude toward Treatment Adherence as adherence precursors.

### Data collection

Three healthcare professionals were trained to interview patients so that the same questions were asked in the same way, and unusual circumstances were handled similarly. Patients were invited to participate when they had an appointment with their family physician, and the interviewers then arranged a home visit in order to complete a questionnaire regarding certain personal characteristics, as well as to count and register the oral hypoglycemic agents. The next day, we reviewed the medical records of each patient and noted their medical prescription. One month later, the interviewers again visited the patients to register the pill count (measure of treatment adherence behaviour), and to ask about their quality of life, knowledge of their medical prescription and their attitude to treatment adherence. The patients were requested to attend the laboratory the next day to examine their glucose levels (fasting glucose and HbA1c).

Treatment Adherence Behaviour (behaviour) was considered a discrete variable and it was analyzed by grouping patients according to the classification proposed by Mason, Matsuyama and Jue [6]. Accordingly, patients were classified with a good behaviour when 90% to 105% of the prescribed medication was taken, whereas patients that took <90% or >105% were classified with a poor behaviour. The percentage adherence was calculated by dividing the difference in the number of pills in the first home visit and the pills remaining at the second home visit by the number of pills prescribed for the interval, and multiplying the result by 100 [6].

Medical Prescription Knowledge (knowledge) was considered a discrete variable, which was measured with the knowledge questionnaire that addressed three issues: the name of the oral hypoglycaemic agents; the dosage; and the dosage frequency. Patients were classified with strong knowledge if their answers agreed with the medical prescription. When at least one response did not agree with the medical prescription we classified these patients with weak knowledge.

Attitude to Treatment Adherence (attitude) was considered a discrete variable and the attitude questionnaire addressed six domains with eleven items. Domains were identified by experts in a thorough search for information about the causes of non-adherence: wellbeing/discomfort (AQ1, AQ2); belief about damage caused by medication (AQ3); the diabetes-treatment complications relationship (AQ4, AQ5); barriers/facilitators to taking medication (AQ6, AQ7); accessibility to healthcare and medical treatment (AQ8, AQ9); and doctor-patient agreements about treatment (AQ10, AQ11). Experts developed eleven questions, two of which were reworded from the Morisky scale [26]. The items were rated on a 5-point Likert scale: 5 = strongly agree, 4 = agree, 3 = indifferent, 2 = disagree, 1 = strongly disagree. The scale was scored by summing the patients' responses and dividing this score by the number of items. If the result was 4 or 5 we classified the patients with a positive attitude, whereas a result between 1 and 3 was classified with negative attitude.

A group of experts used a consensus panel process to generate the items and validate the content. The experts agreed that the content of both questionnaires was valid. The face validity was measured through the identification of ambiguous items by a group of diabetic patients, and minor changes in wording were made based on their comments.

The attitude questionnaire structure was confirmed through a principal component analysis which yielded the same six factors identified by the experts, the eigenvalues were >1 and explained 77.5% of the variance. This analysis supported the construct validity. Moreover, this questionnaire had an acceptable internal consistence (Cronbach's alpha = 0.74).

When the performance of the attitude and knowledge questionnaires was evaluated independently, both questionnaires showed low values of specificity and negative predictive value. These values should be higher in a population with high non-adherence prevalence [27], as in this study.

The attitude and knowledge questionnaires were combined in order to improve their performance through a serial analysis [28]. This analysis considered a patient with positive attitude and strong knowledge as adherent, and a patient with negative attitude or weak knowledge was classified as non-adherent.

The criterion validity was assessed through a performance analysis of the questionnaires, both independently and in a combined manner, taking the pill count (behaviour) as the gold standard. The combination of questionnaires showed the best performance with a specificity of 77.4 % and a negative predictive value of 77.9 %, it identified most of the non-adherent patients.

The questionnaires are shown as an additional file [see Additional file 1], the English version is a translation of the original Spanish questionnaires (available from the first author) that has not been adapted to this language.

Quality of life was assessed by means of the World Health Organization Quality of Life 100 item questionnaire (WHOQOL-100). This was developed by the World Health Organization (WHO) [14] and it consists of six domains: physical, psychological, level of independence, social relationships, environment and spirituality. These domains contain 24 facets, each of which includes four items leading to a total of 96 items. One additional facet of four items pertains to the global quality of life and general health. This facet is not included in the WHOQOL-100 domain structure but it is analyzed as part of the instrument. Each of the facets is summed and each item contributes equally to the facet score and the domain score. All facet scores and domain scores in the WHOQOL-100 are transformed to reflect a scale from 0 to 100, with higher scores reflecting a higher quality of life [29,30]. The original English-language version was translated into Spanish using a method developed by the WHO that included forward and back translation, a bilingual panel of experts and a monolingual panel of diabetics in focus groups. Reliability was assessed by calculating the Cronbach α coefficient (of internal consistency), which was similar to that in other studies and within a range of 0.71 to 0.90 [31].

### Statistical analysis

Descriptive statistics were calculated for the sociodemographic and clinical characteristics of type 2 diabetic patients, for behaviour, and for the combination of knowledge and attitude. Continuous data with a normal distribution were described by the mean and standard deviation, and for data that did not have a normal distribution the median and quartiles were used.

An unpaired Student's t test was used to compare the means of each quality of life domain between binary variables, an analysis of variance (ANOVA) was used as a parametric test of significance for categorical variables, and the Mann-Whitney U was used as non-parametric test of significance when medians had to be compared. Correlations between each quality of life domain and the variables with continuous data were tested with Pearson's r coefficient, when a linear relationship could be confirmed with a scatter plot, otherwise Spearman's rho was used. All results were judged to be statistically significant at the 5 % level (p < 0.05).

A backward multiple linear regression model was constructed to establish the relationship between each quality of life domain and the variables related to adherence, controlling for covariates. For each quality of life domain the initial model included all the variables that were found to be significantly related in the univariate analysis. Variables entered the model with an α ≤ 0.05, and they were removed with an α > 0.10. An adjusted R^2 ^was estimated for the final models to determine the amount of variance in each domain of the quality of life score explained by the predictor variables. Data processing and regression diagnostics used to assess the model assumptions were carried with the STATA statistical software version 9.0 [32].

## Results

When the sociodemographic and clinical characteristics of the patients were analysed, we identified a slightly higher proportion of females in the study (Table 1). The subjects were mostly literate with a basic education level, they were predominantly married and their mean age was 58.7 years. Most of them had hypertension, and they had been prescribed two or three oral hypoglycaemic agents. Patients had a median duration of diabetes of 6 years, and their median fasting glucose was 8.8 mmol/L and the mean HbA1c was 8.8 %.

**Table 1:** Characteristics of diabetic type 2 patients (n = 238).

**CHARACTERISTICS**		
**Sociodemographic characteristics**		
		
**Gender**	**n (%)**	
**Female**		148 (62.2)
**Male**		90 (37.8)
**Educational level**	**n (%)**	
**No education**		20 (8.4)
**Basic education**		169 (71.0)
**Intermediate level**		24 (10.1)
**Higher education**		25 (10.5)
**Marital status**	**n (%)**	
**Singled**		13 (5.5)
**Married/Free union**		182 (76.5)
**Divorced/Widow (er)**		43 (18.1)
**Age**	**mean (SD)**	58.7 (9.6)
**Clinical characteristics**		
**Hypertension**	**n (%)**	
**Yes**		150 (63.0)
**No**		88 (37.0)
**Hypoglycaemic prescription**	**n (%)**	
**Monotherapy**		81 (34.0)
**Polytherapy**		157 (66.0)
**Duration of diabetes in years**		
**(since first diagnosis)**	**median (quartiles)**	6 (3–12)
**Fasting Glucose mmol/L**	**median (quartiles)**	8.8 (6.9–11.9)
**HbA1c %**	**mean (SD)**	8.8 (2.3)
**Adherence**		
**Treatment Adherence Behaviour**	**n (%)**	
**Good**		41 (17.2)
**Poor**		197 (82.8)
**Combination of knowledge and attitude**	**n (%)**	
**Good**		49 (20.6)
**Poor**		189 (79.4)

Only 17.2% of patients showed good treatment adherence behaviour, similar to the results regarding to the precursors of adherence, the combination of knowledge and attitude, where 20.6% of patients displayed both strong knowledge and positive attitude.

The mean scores for each domain of the quality of life scale ranged from 63.1 in the independence domain to 73.9 in the spirituality domain.

The mean quality of life score was significantly higher for male patients than females in the physical, psychological and the environment domain (p < 0.05), but there were no significant differences in the level of independence, social relationship and spirituality domain. With the exception of spirituality, the rest of the domains showed significant differences (p < 0.05) in all categories of educational level, and the mean score in each quality of life domain increased as the educational level also rose. Moreover, patients without hypertension only showed significantly higher scores in the level of the independence domain (p < 0.05). However, there were no significant differences between the mean quality of life scores in any of the six domains between patients with monotherapy and patients receiving more than one oral hypoglycaemic agent (Table 2). There was a significant correlation (p < 0.05) between the level of independence domain and age, as well as with the duration of diabetes. Similarly, the psychological and the environment domain were significantly correlated with the fasting glucose levels (p < 0.05, Table 3).

**Table 2:** Means of the quality of life scores between sociodemographic and clinical characteristics of diabetic type 2 patients.

**SOCIODEMOGRAPHIC AND CLINICAL CHARACTERISTICS**	**QUALITY OF LIFE**					
	
	**Physical**	**Psychological**	**Level of independence**	**Social relationships**	**Environment**	**Spirituality/Religion/Personal beliefs**
	**Mean (SD)**	**Mean (SD)**	**Mean (SD)**	**Mean (SD)**	**Mean (SD)**	**Mean (SD)**
**Gender**						
**Female**	62.4 (17.5)^†^	64.5 (14.6)^†^	62.7 (13.7)	70.0 (12.6)	65.6 (10.6)^†^	74.2 (16.9)
**Male**	67.2 (16.6)^†^	73.1 (10.1)^†^	63.8 (13.4)	72.3 (9.8)	69.6 (8.8)^†^	73.5 (17.3)
**Educational level**						
**No education**	59.6 (21.3)^†^	61.4 (17.2)^†^	58.9 (16.6)^†^	65.0 (12.9)^†^	61.0 (11.7)^†^	71.8 (19.9)
**Basic education**	62.5 (16.4)^†^	66.5 (13.0)^†^	61.9 (12.6)^†^	69.8 (11.1)^†^	66.6 (9.7)^†^	74.3 (15.5)
**Intermediate level**	69.6 (18.0)^†^	73.6 (12.8)^†^	67.9 (15.5)^†^	76.8 (9.8)^†^	69.6 (9.8)^†^	74.2 (24.1)
**Higher education**	74.1 (14.9)^†^	76.0 (11.8)^†^	70.5 (12.1)^†^	77.0 (11.7)^†^	73.5 (8.7)^†^	73.2 (17.3)
**Marital status**						
**Singled**	55.7 (17.0)	61.7 (13.6)	59.8 (12.6)	68.0 (14.0)	66.2 (12.9)	74.0 (18.8)
**Married/Free union**	64.7 (17.2)	68.1 (13.9)	63.4 (13.6)	71.2 (11.6)	67.2 (10.1)	73.6 (17.5)
**Divorced/Widow(er)**	64.7 (17.5)	68.1 (13.0)	63.3 (13.7)	70.3 (11.2)	67.0 (9.7)	75.2 (14.4)
						
**Hypertension**						
**Yes**	63.6 (16.6)	66.7 (13.7)	61.9 (14.3)^†^	70.3 (11.7)	67.3 (10.8)	73.8 (17.1)
**No**	65.2 (18.5)	69.6 (13.6)	65.3 (12.0)^†^	71.9 (11.5)	66.9 (9.0)	74.2 (17.0)
**Hypoglycaemic prescription**						
**Monotherapy**	64.7 (16.5)	68.6 (13.9)	63.8 (14.2)	70.8 (12.1)	66.9 (10.7)	73.6 (19.7)
**Polytherapy**	63.9 (17.7)	67.3 (13.6)	62.8 (13.3)	70.9 (11.4)	67.3 (9.8)	74.1 (15.5)

^†^P < 0.05

**Table 3:** Correlation coefficients between sociodemographic and clinical characteristics of diabetic type 2 patients with quality of life domains.

**SOCIODEMOGRAPHIC AND CLINICAL CHARACTERISTICS**	**QUALITY OF LIFE**					
	
	**Physical**	**Psychological**	**Level of independence**	**Social relationships**	**Environment**	**Spirituality/Religion/Personal beliefs**
Age*	-0.01	0.04	-0.17^†^	-0.03	0.10	0.03
Duration of diabetes in years (since first diagnosis)**	-0.02	0.09	-0.17^†^	-0.02	0.09	0.07
Fasting Glucose mmol/L**	-0.08	-0.15^†^	-0.00	-0.09	-0.18^†^	-0.00
HbA1c %*	-0.05	-0.05	-0.00	-0.00	-0.11	0.00

^†^P < 0.05

* Pearson's r

** Spearman's rho

There were no significant differences between the mean scores in any of the six quality of life domains and the patients' behaviour toward treatment adherence. However, when there was a combination of strong knowledge and a positive attitude, a significant difference was observed in the mean scores of all the domains (Table 4).

**Table 4:** Means in quality of life scores between adherence and its precursors.

**ADHERENCE AND ITS PRECURSORS**	**QUALITY OF LIFE**					
	
	**Physical**	**Psychological**	**Level of independence**	**Social relationships**	**Environment**	**Spirituality/Religion/Personal beliefs**
	**Mean (SD)**	**Mean (SD)**	**Mean (SD)**	**Mean (SD)**	**Mean (SD)**	**Mean (SD)**
**Treatment Adherence Behaviour**						
**Good**	67.5 (16.3)	70.4 (14.9)	64.4 (14.7)	73.0 (11.7)	69.4 (11.2)	74.8 (20.5)
**Poor**	63.5 (17.4)	67.2 (13.4)	62.9 (13.3)	70.4 (11.6)	66.7 (9.8)	73.8 (16.3)
**Combination of knowledge and attitude**						
**Good**	71.5 (15.7)^†^	74.3 (11.3)^†^	66.8 (12.5)^†^	74.9 (9.5)^†^	71.7 (8.1)^†^	78.6 (17.3)^†^
**Poor**	62.3 (17.2)^†^	66.1 (13.8)^†^	62.2 (13.7)^†^	69.8 (11.9)^†^	66.0 (10.3)^†^	72.7 (16.8)^†^

^†^P < 0.05

A multiple linear regression analysis is presented for each of the quality of life domains (Table 5) and it was evident that none of the study variables were associated with the spirituality domain. Patients with a higher education level and a combination of strong knowledge and positive attitude had an increased probability of higher scores in the physical domain, explaining 8% of the variability in the patients' scores. Male patients, with a higher educational level, and a combination of strong knowledge and positive attitude, had a higher probability of achieving greater scores in the psychological domain. This model explained 18 % of the variability in the scores. Patients with a higher level of education, without hypertension, and with a combination of strong knowledge and positive attitude had a greater probability of achieving higher scores at the level of the independence domain, but this probability diminished as the duration of diabetes increased. This model explained 9 % of the variability in the scores. Patients with a higher educational level and a combination of strong knowledge and positive attitude had an increased probability of higher scores in social relationship domain. This model explained 8 % of the variability in the scores. Finally, older male patients with a higher level of education, and a combination of strong knowledge and positive attitude, had a greater probability of achieving higher scores in the environment domain. However, this probability diminished as the glucose levels increased. This model explained 14 % of the variability in the scores.

**Table 5:** Multiple lineal regression analysis of predicting variables for quality of life.

**SOCIODEMOGRAPHIC AND CLINICAL CHARACTERISTICS AND ADHERENCE PRECURSORS**	**QUALITY OF LIFE**											
	
	**Physical**		**Psychological**		**Level of independence**		**Social relationships**		**Environment**		**Spirituality/Religion/Personal beliefs**	
	β *	***P-value***	β	***P-value***	β	***P-value***	β	***P-value***	β	***P-value***	β	***P-value***
**Gender**			0.26	0.00					0.15	0.01		
**Educational level**	0.21	0.00	0.23	0.00	0.21	0.00	0.25	0.00	0.23	0.00		
**Age**									0.17	0.00		
**Hypertension**					0.11	0.05						
**Duration of diabetes in years (since first diagnosis)**					-0.15	0.01						
**Fasting Glucose mmol/L**									-0.13	0.03		
**Combination of knowledge and attitude**	0.19	0.00	0.16	0.00	0.11	0.05	0.14	0.02	0.16	0.00		
												
	*R*^2 ^= 0.09	*R*^2^a = 0.08	*R*^2 ^= 0.20	*R*^2^a = 0.18	*R*^2 ^= 0.10	*R*^2^a = 0.09	*R*^2 ^= 0.09	*R*^2^a = 0.08	*R*^2 ^= 0.15	*R*^2^a = 0.14		

* β standardized regression coefficients.

## Discussion

In this study, treatment adherence behaviour was not associated with any quality of life domain. However, an association was observed when we combined two precursors of adherence, medical prescription knowledge and attitude to treatment adherence, with five of the six domains (physical, psychological, level of independence, social relationship and environmental).

In contrast to previous studies carried out on a Mexican population with type 2 diabetes where 50% of patients were adherent to their treatment [33,34], only 17.2 % of the patients displayed good adherence behaviour here as measured through a pill count. The earlier studies classified patients as adherent if they took ≥ 80% of the medicine prescribed, although this classification method may introduce a bias due to misclassification as indicated by Pullar T [35]. We used a narrower classification scale of 90% to 105% in order to reduce this problem, as suggested by Mason, Matsuyama and Jue [6]. Pill count in this study showed a sensitivity to adherence of 83.3% and a specificity of 89.4%, using electronic monitoring as the gold standard [36]. Co-morbidity may be an important characteristic that could modify adherence behaviour [37-39], and it is common that diabetic patients also suffer hypertension. Indeed, in our study population 63% of the patients suffered hypertension.

Since the criterion validity for the combination of knowledge and attitude was good, we used them as precursors of adherence behaviour. Indeed, they are both easy to apply and cheap, and thus, they can be readily used in daily practice within a public primary health care setting where there are always budget constraints. Both the attitude scale and the knowledge questionnaire showed low sensitivity and specificity, which means that it is not advisable to use them in isolation but rather, they should be used together to obtain valid information regarding patient adherence precursors. We do not have information of studies that have evaluated medical prescription knowledge and attitude toward treatment adherence as adherence precursors, and therefore, we can not make any comparison.

The mean quality of life scores for the different domains ranged from 63.1 to 73.9. We selected patients without diabetic complications and therefore, the quality of life may be overestimated since such complications have an important impact on patient's quality of life. In fact, our study population displayed higher scores in all of the quality of life domains when compared with Bonomi's chronic patients [7] and Lingjiang's hypertensive patients [30]. When our patients were compared with a healthy population [7], they were attributed lower scores in almost all domains, except the social relationships and spirituality domains. Religion is by nature social and Mexican culture has a strong religious burden [40]. Hence, we can explain the higher scores in these domains (social relationships, spirituality) since 96.5% of Mexicans profess some religion and 92% of them are Catholics [41]. Spiritual well being has been suggested as an important internal source that people use to adjust to the problems that a disease such as diabetes may generate [42].

In diabetic patients, quality of life is affected when there are co-morbid conditions [43] or complications [44], and in relation to their treatment, the use of insulin is negatively associated with their quality of life [45]. The association between adherence to oral hypoglycaemic agents and quality of life has been evaluated using different methods to measure adherence. While Cote [19] and Holzemer [17] used self-reporting, Billups [18] and Sung [16] used computerized prescription records. As in our study, none of these studies found an association between adherence and any quality of life domain. Other demographic and psychosocial factors have been associated with quality of life, such as associations between personal attitude of patients and solid specific knowledge of diabetes. Thus, patients with a good, optimistic outlook on life and strong beliefs in self efficacy had a better quality of life score [46]. Indeed, another study found that satisfaction in social relationships and friendships, worry about diabetic complications and treatment satisfaction contributes to the strongest variation in patient's quality of life [47]. In these studies, analyses within specific quality of life domains were made.

Educational level was the only sociodemographic variable associated with five of the six quality of life domains. Although there are contradictory results about this relationship, diabetic patients with higher education may have better social support, positive self esteem, a better understanding of the disease, its treatment and complications [47,48], characteristics that may promote strong knowledge and positive attitude about treatment adherence.

The level of education and the combination of knowledge and attitude were weakly associated with the physical, level of independence and social relationship domains. This association may be explained by the absence of other determinants of the quality of life in the model, such as complications or other psychological variables. Nevertheless these two variables had a stronger association in the psychological and environmental domains, probably due to the fact that these domains take into account the perceptions such as positive experiences, ability to make decisions, and the way patients feel themselves, as well as the patients sense of freedom, security of having financial resources to meet their needs, and the opportunity to acquire new skills and knowledge.

The association between most of the quality of life domains with the combination of knowledge and attitude to treatment adherence abides by the congruity principle of Osgood and Tannenbaum [49]. This can be explained by the "associative assertions" that patients show when they give a positive evaluation in both quality of life (high mean scores) and strong knowledge and positive attitude to treatment adherence, as well as the negative evaluation when the lowest mean scores in terms of weak knowledge and negative attitude toward treatment adherence. Thus, strong knowledge and positive attitude as precursors of good adherence behaviour may promote and improve the quality of life. This explanation is consistent with the model proposed by Rose M et al. [46].

### Limitations

This study had some limitations, such as the fact that the cross-sectional study design ascertained quality of life and adherence at the same time. Although, there was an association between these variables, we do not know its directionality, that is, we do not know if higher scores in quality of life could have caused adherence or if non-adherence could have caused lower scores in quality of life. Besides, since these variables are continuums and they change over time, measurements at only one point in time as taken here have more limited value. Therefore, a longitudinal study would provide a more complete picture regarding which of these variables could predict the other, controlling for exposure changes. Since participants were enrolled in primary care and lived in a city, the results can not be easily transferred to all diabetic patients. Furthermore, some authors have mentioned that home visits may influence medication-taking [6] causing an overestimation of the results. Nevertheless, we realized that it was important to visit patients because most of them had more medications at home than the ones they received in their last medical consultation. Thus, in this way we were able to register all their tablets. Moreover, adherence magnitude in this study was lower than other studies that have used a different measurement method [37]. Finally, complications or insulin prescription are known confounders, since these variables are associated with an impairment in quality of life [44,45] and with lower adherence [37,50]. An accepted strategy to control confounders is restriction and therefore, we did not include patients with complications or those that were prescribed insulin.

## Conclusion

In this study, we identified an association between most quality of life domains and the combination of medical prescription knowledge and attitude toward treatment adherence in type 2 diabetic patients. Based on these results, we consider it important to explore psychological precursors of treatment adherence behaviour in type 2 diabetic patients and to carry out interventions that change negative attitude toward treatment adherence and promote medical prescription knowledge, which may help to improve the quality of life of such patients.

## Competing interests

The authors declare that they have no competing interests.

## Authors' contributions

YVM and CAP–A participated in the study design, in the acquisition of data, the statistical analysis and interpretation of data, and in the drafting of the manuscript. RAR–P contributed to the design of the study, the statistical analysis and interpretation of the data, and the drafting of the manuscript. JJV–M collaborated in the data acquisition and the drafting of manuscript.

## Pre-publication history

The pre-publication history for this paper can be accessed here:



## Supplementary Material

Additional file 1 Questionnaires of adherence behaviour precursors. The questionnaires provided represent the two adherence behaviour precursors (Medical Prescription Knowledge and Attitude toward Treatment Adherence). Click here for file

## References

[B1] Feinstein AR (1990). On white-coat effects and the electronic monitoring of compliance.. Arch Intern Med.

[B2] UKPDS (1998). Intensive blood-glucose control with sulphonylureas or insulin compared with conventional treatment and risk of complications in patients with type 2 diabetes (ukpds 33). Lancet.

[B3] UKPDS (1998). Effect of intensive blood-glucose control with metformin on complications in overweight patients with type 2 diabetes (ukpds 34). Lancet.

[B4] Bergman U, Wilholm BE (1981). Drug-related problems causing admission to a medical clinic. European Journal of Clinical Pharmacology.

[B5] Cramer JA (2004). A systematic review of adherence with medications for diabetes. Diabetes Care.

[B6] Mason BJ, Matsuyama JR, Jue SG (1995). Assessment of sulfonylurea adherence and metabolic control. Diabetes Educator.

[B7] Bonomi AE, Patrick DL, Bushnell DM, Martin M (2000). Validation of the United States' version of the World Health Organization Quality of Life (WHOQOL) instrument.. Journal of Clinical Epidemiology.

[B8] Testa MA, Simonson DC (1998). Health economic benefits and quality of life during improved glycemic control in patients with type 2 diabetes mellitus: a randomized, controlled, double-blind trial.[see comment]. JAMA.

[B9] Pibernik-Okanovi M (2001). Psychometric properties of the World Health Organisation quality of life questionnaire (WHOQOL-100) in diabetic patients in Croatia. Diabetes Research & Clinical Practice.

[B10] Lau CY, Qureshi AK, Scott SG (2004). Association between glycaemic control and quality of life in diabetes mellitus. J Postgrad Med.

[B11] Weinberger M, Kirkman MS, Samsa GP, Cowper PA, Shortliffe EA, Simel DL, Feussner JR (1994). The relationship between glycemic control and health-related quality of life in patients with non-insulin-dependent diabetes mellitus. Med Care.

[B12] Goddijn PP, Bilo HJ, Feskens EJ, Groeniert KH, van der Zee KI, Meyboom-de Jong B (1999). Longitudinal study on glycaemic control and quality of life in patients with Type 2 diabetes mellitus referred for intensified control. Diabetic Medicine.

[B13] Kleefstra N, Ubink - Veltmaat LJ, Houweling ST, Groenier KH, Meyboom- de Jong B, Bilo HJ (2005). Cross- sectional relationship between glycaemic control, hyperglycaemic symptoms and quality of life in type 2 diabetes (ZODIAC-2). Neth J Med.

[B14] Pitale S, Kernan-Schroeder D, Emanuele N, Sawin C, Sacks J, Abraira C (2005). Health-related quality of life in the VA Feasibility Study on glycemic control and complications in type 2 diabetes mellitus. J Diabetes Complications.

[B15] Pippalla RS, Chinburapa V, Duval R, Akula RS (1997). Interrelationships of quality of life, compliance, clinical outcomes and life satisfaction: a cross-sectional study on hypertensive geriatrics. Journal of Clinical Pharmacy and Therapeutics.

[B16] Sung JC, Nichol MB, Venturini F, Bailey KL, McCombs JS, Cody (1998). Factors affecting patient compliance with antihyperlipidemic medications in an HMO population. Am J Manag Care.

[B17] Holzemer WL, Corless IB, Nokes KM, Turner JG, Brown MA, Powell-Cope GM, Inouye J, Henry SB, Nicholas PK, Portillo CJ (1999). Predictors of self-reported adherence in persons living with HIV disease. AIDS Patient Care & Stds.

[B18] Billups SJ, Malone DC, Carter BL (2000). The relationship between dug therapy noncompliance and patient characteristics, health-related quality of life, and health care costs. Pharmacotherapy.

[B19] Cote I, Farris K, Feeny D (2003). Is adherence to drug treatment correlated with health-related quality of life?. Quality of Life Research.

[B20] WHOQOL GROUP (1997). Measuring Quality of Life.

[B21] Haynes RB, Press JHU (1979). Determinants of compliance: The disease and the mechanics of treatment.. Adherence to Long-Term Therapies Evidence For Action.

[B22] Rand CS (1993). Measuring adherence with therapy for chronic diseases: implications for the treatment of heterozygous familial hypercholesterolemia.. Adherence to Long-Term Therapies: Evidence for Action.

[B23] WHO (2003). Adherence to Long-Term Therapies. Evidence For Action.

[B24] Gerber KE, Nehemkis AM (1986). Compliance: the dilemma of the chronically ill.

[B25] Ajzen I, Fishbein M (2000). Attitudes and the attitude-behavior relation: Reasoned and automatic processes. European Review of Social Psychology.

[B26] Morisky DE, Green LW, Levine DM (1986). Concurrent and predictive validity of a self-reported measure of medication adherence. Med Care.

[B27] Sackett DL, Haynes RB, Guyatt GH, Tugwell P (1991). Clinical Epidemiology: a Basic Science for Clinical Medicine.

[B28] Fletcher RH, Fletcher SW, Wagner EH (1996). Clinical Epidemiology. The Essentials.

[B29] WHOQOL GROUP (1995). The World Health Organization Quality of Life assessment (WHOQOL): position paper from the World Health Organization.. Soc Sci Med.

[B30] Lingjiang LI, Derson Y, Shuiyuan X, al (2004). Psychometric properties of the WHO Quality of life questionnaire (WHOQOL-100) in patients with chronic diseases and their caregivers in China.. Bull World Health Organ.

[B31] WHOQOL G, Power M, Kuyken W, Orley J (1998). The World Health Organization Quality of Life Assessment (WHOQOL): development and general psychometric properties.. Soc Sci Med.

[B32] Corporation S (2005). Stata Statistical Software: Release 9.0.

[B33] Duran-Varela BR, Rivera-Chavira B, Franco-Gallegos E (2001). Phamacological therapy compliance in diabetes. Salud Publica Mex.

[B34] Hernandez-Ronquillo L, Tellez-Zenteno JF, Garduno-Espinosa J, Gonzalez-Acevez E (2003). Factors associated with therapy noncompliance in type-2 diabetes patients. Salud Publica Mex.

[B35] Pullar T, Kumar S, Tindall H, Feely M (1989). Time to stop counting the tablets?. Clin Pharmacol Ther.

[B36] Martinez YV, Prado-Aguilar CA, Rascon-Pacheco RA, Segovia-Bernal Y (2006). Validity of three different methods to measure treatment adherente in type 2 diabetics. XV National Meeting of Health Research.

[B37] Donnan PT, MacDonald TM, Morris AD (2002). Adherence to prescribed oral hypoglycaemic medication in a population of patients with Type 2 diabetes: a retrospective cohort study. Diabet Med.

[B38] MacLaughlin EJ, Raehl CL, Treadway AK, Sterling TL, Zoller DP, Bond CA (2005). Assessing medication adherence in the elderly: which tools to use in clinical practice?. Drugs Aging.

[B39] Ciechanowski PS, Katon WJ, Russo JE (2000). Depression and diabetes: impact of depressive symptoms on adherence, function, and costs. Arch Intern Med.

[B40] Rivera-Ledesma A, Montero M (2005). Espiritualidad y religiosidad en adultos mayores mexicanos. Salud Mental.

[B41] INEGI (2000). Instituto Nacional de Estadistica Geografia e Informatica: XII Censo Nacional de Población y Vivienda..

[B42] Landis BJ (1996). Uncertainty, spiritual well-being, and psychosocial adjustment to chronic illness. Issues Ment Health Nurs.

[B43] Rejeski WJ, Lang W, Neiberg RH, Van Dorsten B, Foster GD, Maciejewski ML, Rubin R, Williamson DF (2006). Correlates of health-related quality of life in overweight and obese adults with type 2 diabetes. Obesity (Silver Spring).

[B44] (1999). Quality of life in type 2 diabetic patients is affected by complications but not by intensive policies to improve blood glucose or blood pressure control (UKPDS 37). U.K. Prospective Diabetes Study Group. Diabetes Care.

[B45] Redekop WK, Koopmanschap MA, Stolk RP, Rutten GE, Wolffenbuttel BH, Niessen LW (2002). Health-related quality of life and treatment satisfaction in Dutch patients with type 2 diabetes. Diabetes Care.

[B46] Rose M, Fliege H, Hildebrandt M, Schirop T, Klapp BF (2002). The network of psychological variables in patients with diabetes and their importance for quality of life and metabolic control. Diabetes Care.

[B47] Varghesea RT, Salinia R, Abrahama P, Reeshmaa KK, Vijayakumar K (2007). Determinants of the quality of life among diabetic subjects in Kerala, India. Diabetes & Metabolic Syndrome: Clinical Research & Reviews.

[B48] Ranchor AV, Bouma J, Sanderman R (1996). Vulnerability and social class: differential patterns of personality and social support over the social classes. Pers Individ Dif.

[B49] Osgood CE, Tannenbaum PH (1955). The principle of congruity in the prediction of attitude change. Psychol Rev.

[B50] Rubin RR (2005). Adherence to pharmacologic therapy in patients with type 2 diabetes mellitus. Am J Med.

