# Supplementary material for: Quality of life associated with treatment adherence in patients with type 2 diabetes: a cross-sectional study
Source: BMC Health Serv Res. 2008 Jul 30;8:164. doi: 10.1186/1472-6963-8-164 (PMC2519069; doi:10.1186/1472-6963-8-164)
Supplement: Additional file 1 — Questionnaires of adherence behaviour precursors. The questionnaires provided represent the two adherence behaviour precursors (Medical Prescription Knowledge and Attitude toward Treatment Adherence). [file 1472-6963-8-164-S1.doc]

**Medical Prescription Knowledge Questionnaire.**

1. From the medical record of the diabetic patient, the family physicians prescription must be identified. Check (**√**) the corresponding box if the statement corresponds to the information written in the medical record of a diabetic patient.

Medication prescribed A: Glibenclamide dosage:

1 tablet at each intake

2 tablets at each intake

Glibenclamide dosage frecuency:

Medication once a day:

Medication twice a day:

Medication three times a day:

Medication prescribed B: Metformin dosage:

1 tablet at each intake:

2 tablets at each intake:

Metformin dosage frecuency:

Medication once a day:

Medication twice a day:

Medication three times a day:

1. With the following three questions we want to know how you take the medication prescribed by your family physician at the last medical visit. If the patient´s answer corresponds with the information listed in the last section check (**√**) the corresponding box of the statement, otherwise place an (**x**). If the medication is not prescribed leave the box blank.

KQ. 1. What is the name of the diabetes medication prescribed by your family physician?

Medication A: Glibenclamide:

Medication B: Metformin:

KQ. 2. How many tablets a day do you have to consume at each intake?

Medication A: Glibenclamide:

1 tablet at each intake:

2 tablets at each intake:

Medication B: Metformin:

1 tablet at each intake:

2 tablets at each intake:

KQ. 3. How many times a day do you have to take your medication?

Medication A: Glibenclamide:

Once a day:

Twice a day:

Three times a day:

Medication B: Metformin:

Once a day:

Twice a day:

Three times a day:

**Attitude Towards Treatment Adherence Questionnaire.**

For each of the following questions we invite you to respond as best represents your view towards the statement presented in the question.

With the following 11 questions we want to know to what extent do you agree or disagree with the situation that helps or limits diabetic patients with their treatment compliance as indicated by physicians

|  | Strongly agree | Agree | Neither agree or disagree | Disagree | Strongly disagree | Score |
| --- | --- | --- | --- | --- | --- | --- |
| AQ. 1 If diabetic patients felt well, they would stop taking their medications. (-) | 1 | 2 | 3 | 4 | 5 |  |
| AQ. 2 Diabetic patients will get sicker if they stop taking their medications (+) | 5 | 4 | 3 | 2 | 1 |  |
| AQ. 3 The medications given to diabetic patients will cause blindness (-) | 1 | 2 | 3 | 4 | 5 |  |
| AQ. 4 Diabetes is a disease that causes health complications  (+) | 5 | 4 | 3 | 2 | 1 |  |
| AQ. 5 Medications to treat Diabetes will prevent or delay complications associated with Diabetes (+) | 5 | 4 | 3 | 2 | 1 |  |
| AQ. 6 It is difficult for diabetic patients to take their medications at work (-) | 1 | 2 | 3 | 4 | 5 |  |
| AQ. 7 It is advisable that the family members of diabetic patients facilitate their intake of medications (+) | 5 | 4 | 3 | 2 | 1 |  |
| AQ. 8 Diabetic patients have problems complying with their treatment if they live far from the clinic (-) | 1 | 2 | 3 | 4 | 5 |  |
| AQ. 9 Diabetic patients have problems complying with their treatment due to lack of money (-) | 1 | 2 | 3 | 4 | 5 |  |
| AQ. 10 Physicians and diabetic patients should agree on the prescription to treat their Diabetes (+) | 5 | 4 | 3 | 2 | 1 |  |
| AQ. 11 Do you agree with your diabetes treatment? (+) | 5 | 4 | 3 | 2 | 1 |  |
| **TOTAL SCORE** |  | | | | |  |
